# Single-Sided Digital Microfluidic (SDMF) Devices for Effective Coolant Delivery and Enhanced Two-Phase Cooling

**DOI:** 10.3390/mi8010003

**Published:** 2016-12-24

**Authors:** Sung-Yong Park, Youngsuk Nam

**Affiliations:** 1 Department of Mechanical Engineering, National University of Singapore, Block EA, #07-08, 9 Engineering Drive 1, Singapore 117576, Singapore; 2 Department of Mechanical Engineering, Kyung Hee University, 1732 Deokyoungdaero, Yongin 17104, Korea

**Keywords:** digital microfluidics, electrowetting, droplets, micro-scale cooling

## Abstract

Digital microfluidics (DMF) driven by electrowetting-on-dielectric (EWOD) has recently been attracting great attention as an effective liquid-handling platform for on-chip cooling. It enables rapid transportation of coolant liquid sandwiched between two parallel plates and drop-wise thermal rejection from a target heating source without additional mechanical components such as pumps, microchannels, and capillary wicks. However, a typical sandwiched configuration in DMF devices only allows sensible heat transfer, which seriously limits heat rejection capability, particularly for high-heat-flux thermal dissipation. In this paper, we present a single-sided digital microfluidic (SDMF) device that enables not only effective liquid handling on a single-sided surface, but also two-phase heat transfer to enhance thermal rejection performance. Several droplet manipulation functions required for two-phase cooling were demonstrated, including continuous droplet injection, rapid transportation as fast as 7.5 cm/s, and immobilization on the target hot spot where heat flux is locally concentrated. Using the SDMF platform, we experimentally demonstrated high-heat-flux cooling on the hydrophilic-coated hot spot. Coolant droplets were continuously transported to the target hot spot which was mitigated below 40 K of the superheat. The effective heat transfer coefficient was stably maintained even at a high heat flux regime over ~130 W/cm^2^, which will allow us to develop a reliable thermal management module. Our SDMF technology offers an effective on-chip cooling approach, particularly for high-heat-flux thermal management based on two-phase heat transfer.

## 1. Introduction

Digital microfluidics (DMF) is a liquid-handling technology driven by the electrowetting-on-dielectric (EWOD) principle, which modifies the surface tension of a liquid droplet on a solid surface with an applied electric field [1,2,3]. By addressing digitized electrodes, DMF rapidly manipulates liquid droplets in the volume ranging from pL to µL and performs various drop-wise functions such as injection, transportation, merging, mixing, and cutting [4,5,6,7]. Due to the benefits of the surface tension force dominance over body forces in micro/mesoscales, fast response time in the range of milliseconds, and low-power consumption, the DMF technology has been used for numerous applications, including lab-on-a-chip [8,9,10,11,12], electronic papers [13,14,15], tunable optical components [16,17,18], and energy harvesting [19,20,21].

In recent years, DMF has drawn attraction as a liquid-handling platform for effective cooling of target heating sources [22,23,24,25,26,27,28]. Unlike conventional cooling technologies such as heat pipes [29,30], heat spreaders [31,32], microchannel-guided liquid cooling [33,34], and jet impingement cooling [35], DMF does not require additional mechanical components such as pumps, valves, microchannels, and capillary wick structures for coolant delivery. Instead, it utilizes the EWOD-based surface tension force (i.e., non-mechanical) to rapidly deliver coolant liquid in a discrete manner, while requiring extremely low power consumption in the range of mW. Furthermore, it just consists of several thin layers of a dielectric and an electrode on top of heating sources. Such thin layers (a few μm) between coolant liquid and the heating source enhance heat dissipation performance by reducing the thermal resistance.

**Figure 1:**
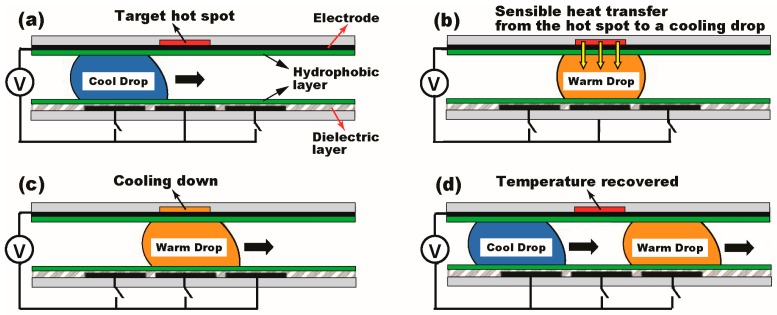
A hot spot cooling approach based on digital microfluidic (DMF) technology. (**a**) Cooling liquid is sandwiched between the top and bottom plates coated with a hydrophobic layer; (**b**,**c**) By sequentially addressing digitized electrodes, a droplet is rapidly transported to the target heating source (i.e., hot spot) where the heat is transferred from the hot spot to the cooling droplet and the temperature lowers down; (**d**) Then, the droplet leaves for a heat sink to release the heat to outside. These processes are rapidly repeated to keep mitigating the hot spot temperature. The DMF-based cooling method mainly uses sensible heat transfer for thermal rejection from the hot spot. However, such a single-phase cooling approach limits the level of applicable heat flux.

Figure 1 presents schematics of the hot spot cooling using a typical sandwiched DMF platform. A cooling droplet is sandwiched between two parallel plates with a few hundreds of μm gap and transported to the hydrophobic-coated hot spot (Figure 1a). Upon the arrival, the droplet experiences sensible heat transfer to take out the heat from the target hot spot (Figure 1b), thus cooling down the temperature (Figure 1c). Then, the droplet leaves for a heat sink to release the heat to outside, while the hot spot temperature is recovered (Figure 1d). To continuously mitigate the hot spot temperature, these processes are rapidly repeated with a series of the droplets. Using this DMF-based cooling approach, Paik et al. demonstrated thermal mitigation of the target hot spot on which a water droplet passed through [25]. The hot spot was initially heated up to 90 °C at the heat flux of 33.3 W/cm^2^. Upon arrival of the cooling droplet, the temperature at the hot spot dropped down to 60 °C and recovered to the original temperature after its departure. Whilst increasing the frequency of the droplet delivery, the target hot spot was maintained at low temperature [27]. Since sandwiched DMF devices typically use sensible heat transfer as the main thermal rejection mechanism, it was further proposed to utilize liquid metals or alloys as coolant which offers a few orders of magnitude higher thermal conductivity than that of water to improve heat transfer capability [22]. Analytical studies were also conducted to support the fact that the droplet transportation between two parallel plates causes the circulation flow inside of the droplet and hence significantly enhances heat transfer performance [23]. 

Despite numerous studies of DMF-based cooling previously reported [22,23,24,25,26,27,28], such a single-phase heat transfer approach cannot be used for high-heat-flux (>100 W/cm^2^) applications, for example, high power density semiconductors such as graphic processing units (GPUs), power amplifiers, and insulated gate bipolar transistors (IGBTs). In order to achieve high-heat-flux dissipation, the two-phase heat transfer mechanism needs to be integrated [29,30,34]. However, DMF devices could not incorporate the two-phase cooling mechanism due to the difficulties associated with active boiling and evaporation within the tightly sandwiched configuration (a few hundreds of μm gap) and less droplet pinning effect on the hydrophobic-coated surface of the hot spot.

**Figure 2:**
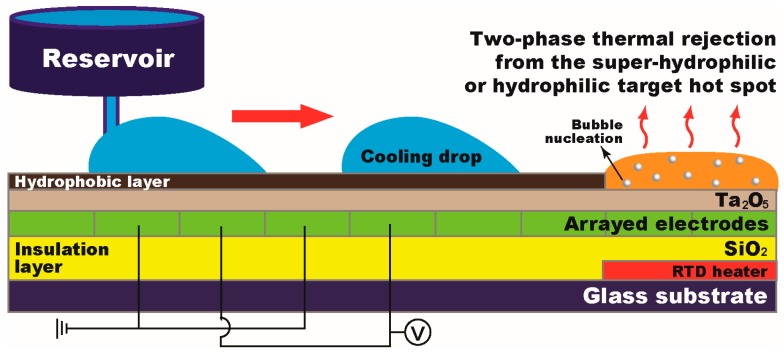
A schematic of the single-sided digital microfluidic (SDMF) device and the working principle of effective liquid delivery and two-phase cooling. A cooling droplet is continuously injected and transported to the target hot spot on which the surface is modified to super-hydrophilic or hydrophilic to enhance the heat rejection capability of the device. The target hot spot experiences two-phase heat transfer while coolant liquid is vaporized and boiled. The device consists of multiple layers on a glass substrate (not to scale): a 100 nm resistance thermal detector (RTD), a 1.5 μm SiO_2_ for passivation, an array of the 1 μm Au electrodes, a 120 nm Ta_2_O_5_ as a dielectric layer, and a 40 nm FluoroPel polymer as a hydrophobic layer.

In this paper, we present a single-sided digital microfluidic (SDMF) device, shown in Figure 2, which enables not only effective coolant delivery without additional mechanical components such as pumps, capillary wicks, and microchannels, but also two-phase evaporation/boiling heat transfer on the target hot spots with the minimal volume of cooling liquid on a single-sided plate. Cooling droplets can be continuously injected, transported as fast as 7.5 cm/s, and immobilized on the hydrophilic hot spot surface. Hot spot surface properties were investigated to enhance the thermal rejection capability of the device. Using the SDMF platform, we experimentally demonstrated high-heat-flux cooling on the hydrophilic-coated hot spot. Coolant droplets were transported to the target heating location which was mitigated below 40 K of the superheat (i.e., the difference between the surface temperature and saturation temperature). The effective heat transfer coefficient, which is estimated as the heat flux divided by the superheat, was maintained stable even at a high heat flux regime over ~130 W/cm^2^, which will allow us to develop a reliable thermal management module. Our SDMF technology potentially offers an effective on-chip cooling approach particularly for high-heat-flux thermal management based on two-phase heat transfer.

## 2. Device Fabrication and Working Principle

Figure 2 illustrates a schematic of the SDMF device and its working principle for coolant delivery and two-phase cooling. For the on-chip heat transfer study, we used the resistance thermal detector (RTD) which is able to directly measure the temperature of a target hot spot. Fabrication of SDMF devices began with the RTD embedded in the device. A gold (Au) layer of 100 nm thickness was first deposited on a glass substrate by a sputter and patterned for the RTD fabrication. On top of the RTD pattern, a 1.5 µm thick layer of silicon dioxide (SiO_2_) was subsequently deposited as an insulation layer via plasma enhanced chemical vapor deposition (PECVD), on which an array of the 1 µm thick Au electrodes was deposited and patterned with the 1.45 mm pitch and the 20 µm spacing. Subsequently, a dielectric layer of a tantalum pentoxide (Ta_2_O_5_) was deposited in 120 nm thickness by an electron beam evaporator. To provide a hydrophobic surface required for EWOD-based liquid delivery, a FluoroPel polymer solution (FluoroPel PFC 1601V, Cytonix) was spin-coated at 3000 rpm for 30 s, followed by being baked on a hot plate at 180 °C for 30 min. To provide a hydrophilic surface property on the target hot spot, a Kapton tape was simply used to cover the only hot spot area with the size of 4 × 4 mm^2^. After spin-coating and curing of a FluoroPel polymer solution, the tape was removed and thus the surface of the Ta_2_O_5_ layer was directed exposed to provide a hydrophilic hot spot surface. The RTD also functioned as a thin-film heater to simulate a heating source on the target hot spot. Our preliminary measurement and calibration of the RTDs indicated an almost linear relationship between the electrical resistance of an Au thermometer and the temperature change of a heating source.

Manipulation of coolant liquid on SDMF is based on the EWOD principle, which controls the surface tension of a liquid droplet on a solid surface. An applied electric potential re-distributes the charge at the liquid–solid interface and decreases the associated interfacial energy by expanding the surface area of the droplet. The droplet contact angle is consequently reduced from θ_0_ to θ. The Young–Lippmann equation mathematically describes such a contact angle change with respect to the voltage drop (*V*) across the dielectric layer [36]:

(1) $$\cos\theta =\cos\theta_{0}+\frac{1}{2\gamma }\frac{\varepsilon_{0}\varepsilon_{r}}{t}V^{2}$$

where γ is the surface tension between the droplet and surrounding medium, ε_0_ is the vacuum permittivity, ε*_r_* is the dielectric constant, and *t* is thickness of the dielectric layer. When a digitized electrode nearby the droplet is electrically addressed, the contact angle changes at one of the edges and such a droplet shape change causes the pressure gradient inside the droplet. As a result, the droplet is attracted to the electrode addressed. As shown in Figure 2, a cooling droplet is transported to the target hot spot by sequentially addressing digitized electrodes. Upon the arrival, the droplet experiences two-phase heat transfer (e.g., evaporation and boiling) on a hydrophilic surface of the hot spot. These steps are rapidly repeated to achieve continuous thermal modulation on the target heating location before the droplet dries out.

## 3. Droplet Manipulation on a Single-Sided Surface

To achieve effective two-phase cooling on SDMF, we built up a LabVIEW-based electric control system that enables the regulation of the coolant droplet volume injected and dispensing frequency. Two adjacent electrodes are simultaneously activated: one is powered and another is grounded. The next pair of the electrodes is sequentially activated to transport droplets on a single-sided surface. With this electric control scheme, we have achieved several important droplet manipulation functions necessary for two-phase heat transfer, including continuous droplet injection, rapid transportation, and immobilization on top of the target hot spot. 

**Figure 3:**
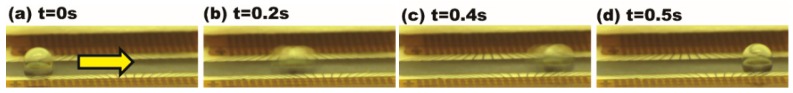
Video snapshots showing the transportation of a 30 µL water droplet on a single-sided surface without complex mechanical components. A transportation speed was as fast as 7.5 cm/s at a given voltage of 35 V. (**a**) *t* = 0 s; (**b**) *t* = 0.2 s; (**c**) *t* = 0.4 s; (**d**) *t* = 0.5 s.

Figure 3 shows the droplet transportation on a single-sided surface without additional mechanical components such as pumps, valves, microchannels, and capillary wicks. A 30 μL water droplet was transported as fast as 7.5 cm/s at a 35 V DC bias and an interval of 20 ms between each activation of the paired electrodes (supplementary movie 1). At a lower voltage of 20 V, the droplet was not actuated. The reduced electric potential could not provide a large enough EWOD force to overcome the contact angle hysteresis [37]. Another test was also conducted with the faster interval of 10 ms. It was observed that the droplet was just transported along a couple of the electrodes, but not all the patterned 24 electrodes. This might be because the electrode activation is too fast for the droplet to immediately follow it up.

**Figure 4:**
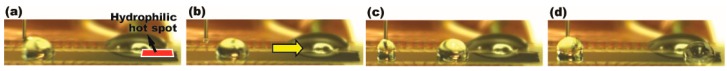
Video snapshots showing a complete set of the droplet manipulations to achieve effective two-phase cooling on SDMF. A 12.5 μL water droplet is injected, directly transported, and immobilized on the hydrophilic hot spot. The droplet expands the contact area and increases the heat transfer rate on the hydrophobic surface. A 35 DC bias was applied. (**a**) Before; and (**b**) after the droplet injection, *t* = 0 s; (**c**) *t* = 3.2 s; and (**d**) *t* = 6.5 s.

Compared to droplet transportation, the injection or cutting process is more challenging on a single-sided plate than a sandwiched one. According to the previous study [4], the tight constraint of the droplet height is critical to achieve droplet cutting. In DMF devices, the droplet height is typically confined by the top and bottom plates with a few hundreds of μm gap, while such a height constraint cannot be permitted on a single-sided configuration. To facilitate the droplet injection process on a single-sided surface, our group previously used a pin connection to a reservoir, which was able to reduce the surface tension force by constraining the droplet area at the pin tip [7]. Using this method, light-driven droplet injection at 2.5 µL was demonstrated with less than 1% volume variation. This approach was similarly used to control the injected droplet volume as well as feeding frequency. Figure 4 shows a complete set of the droplet-based functions that are necessary for two-phase cooling on SDMF (supplementary movie 2). Cooling liquid is pumped through a pin from the reservoir by EWOD forces and the liquid grows to touch the bottom surface (Figure 4a). The surface tension force of the droplet is minimized at the tiny pin tip to facilitate the droplet cutting process. By electrically addressing the nearby electrodes, the EWOD force takes off the droplet from the pin tip (Figure 4b). Subsequently, it is rapidly transported and immobilized on the hydrophilic surface of the target hot spot where the droplet contact area increases to enhance the heat transfer rate (Figure 4d). Meanwhile, the next droplet is successively dispensed to continuously mitigate the hot spot temperature. 

## 4. Experimental Results and Discussion

### 4.1. Enhaced Two-Phase Heat Transfer on SDMF

**Figure 5:**
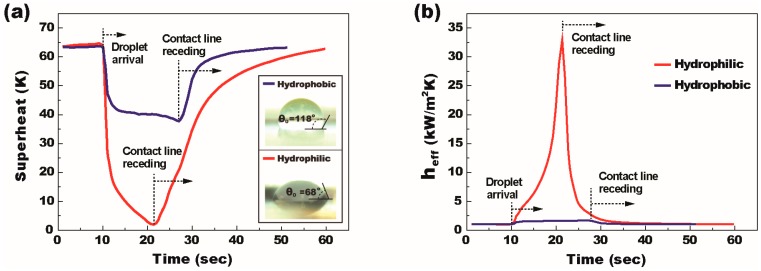
The SDMF’s capability for two-phase heat transfer in terms of (**a**) superheat; and (**b**) effective heat transfer as a function of time. A 5 μL water droplet was injected on a SDMF platform and transported to hydrophobic- and hydrophilic-coated hot spots, which were initially heated up to around 164 °C at the heat flux of 6.5 W/cm^2^ and then thermally mitigated by the cooling liquid. The insets show the contact angle of a water droplet on each of the surfaces.

Recent studies reported that a thin layer of cooling liquid on a super-hydrophilic or hydrophilic surface is able to enhance heat transfer performance by reducing thermal resistance of the liquid layer [38,39,40]. This low-energy surface is also free from the hydrophobic polymer coating that behaves as an additional thermal barrier. However, DMF-based cooling studies [22,23,24,25,26,27,28] previously reported critically require the hydrophobic polymer coating on top of the hot spot to facilitate droplet transportation. As indicated in Figure 1, while a cooling droplet passes over the target hot spot, the heat is rejected based on sensible heat transfer, which limits the level of applicable heat flux. Unlike such a limited heat transfer performance on DMF, a single-sided configuration of SDMF devices allows two-phase heat transfer by forming a thin layer of a coolant droplet on the super-hydrophilic or hydrophilic surface of the hot spot and thus can be used for high-heat-flux applications, for example, high power density semiconductors such as GPUs, power amplifiers, and IGBTs. Hence, our first study was conducted to see the thermal performance relying on the hot spot surface conditions, for which we prepared two SDMF devices: one with a hydrophobic surface provided by spin-coating of a FluoroPel polymer on the hot spot and another with a hydrophilic surface by removing the polymer layer (i.e., a coolant droplet directly contacts on the Ta_2_O_5_ surface). De-ionized water was selected as coolant liquid for the heat transfer study and the follow-up wettability tests show the contact angle at 118° on the hydrophobic surface, while 68° on the hydrophilic surface (see the insets of Figure 5a).

To quantify the SDMF’s capability for two-phase heat transfer, thermal experiments were conducted within an enclosed environment, assuming the near-saturation condition. The superheat was calculated by the temperature difference between the hot spot temperature and the saturation temperature (100 °C) at 1 atm operating pressure and the heat flux was defined as the supplied power divided by the area of the micro heater. The effective heat transfer coefficient (*h*_eff_) was then defined as the heat flux divided by the superheat. The hot spot temperature was measured from the electrical resistance change of the RTD embedded in the device. Since the layers’ thickness was as thin as ~2.5 μm, we reasonably ignored the heat laterally spreading through the thin film layers deposited on top of the heater. The bottom side of the substrate was thermally insulated by attaching a thick glass wool. 

The wall superheat initially reached 64 K with the heat flux of 6.5 W/cm^2^ for both hydrophobic and hydrophilic hot spots. As presented in Figure 2, the SDMF platform allowed the injection of a 5 μL droplet and its transportation to the target hot spot for two-phase thermal mitigation. Figure 5a,b shows the effects of the hot spot surface conditions on the wall superheat and the effective heat transfer coefficient, respectively. When the droplet arrived at the hot spot, the superheat rapidly decreased due to the increase in the heat transfer coefficient associated with the droplet evaporation. In the beginning stage, the heat transfer coefficient rapidly increases as the evaporation becomes more active and the conduction resistance within the droplet decreases as the droplet height decreases. When the contact line of the droplet starts receding due to evaporation, the droplet contact area decreases and the superheat is rapidly recovered back to the initial value. Although the overall tendency of the superheat looks similar for both cases, the heat transfer performance is significantly different according to the surface wettability on the hot spot area. For the hydrophobic-coated hot spot, which is the typical situation of the DMF devices, the maximum temperature drop and the effective heat transfer coefficient were observed to be ~25 K and 1.7 kW/m^2^K, respectively. For the hydrophilic hot spot, both the maximum temperature drop and the effective heat transfer coefficient were significantly enhanced and measured to be as high as ~62 K and 33 kW/m^2^K (almost 20 times larger than that of the hydrophobic), respectively. Due to the enhanced evaporation rate, the duration of droplet evaporation on the hydrophilic hot spot was ~65% shorter than that of the hydrophobic-coated one.

The significantly enhanced heat transfer performance on the hydrophilic hot spot can be explained by the following reasons. First, due to the low contact angle hysteresis and the resulting low contact line pinning on the hydrophobic surface, the cooling droplet skittered away from the hydrophobic hot spot and thus the hot spot area was not fully covered by the cooling liquid, while the droplet firmly adhered to the hydrophilic hot spot and maintained a relatively large contact area until the contact line receding occurred. Second, the thermal conduction resistance through a 5 μL water droplet placed on the hydrophilic hot spot (*R*_cond_ = 98.4 K/W) is much lower than that on the hydrophobic hot spot (*R*_cond_ = 273.7 K/W). The conduction resistance was calculated from the method of integration of the temperature difference between two neighboring isothermal surfaces, $R_{\mathrm{cond}}=\theta /\left(4\pi rk_{w}\sin\theta \right)$ where θ, *r*, and *k_w_* represent the contact angle, a radius of droplet, and thermal conductivity of cooling liquid, respectively [41,42]. Finally, the droplet on the hydrophilic hot spot has a larger contact area of thin evaporative film compared to the one on the hydrophobic spot, which provides a significantly higher evaporation rate, especially near the droplet contact line. The experimental results in Figure 5 clearly show the SDMF’s capability of two-phase cooling on a hydrophilic hot spot surface, which cannot be provided by conventional DMF devices only allowing sensible heat transfer on a hydrophobic hot spot surface. To further improve the thermal performance, a super-hydrophilic surface on top of the hot spot is suggested for SDMF devices.

### 4.2. Continuous Thermal Mitigation

**Figure 6:**
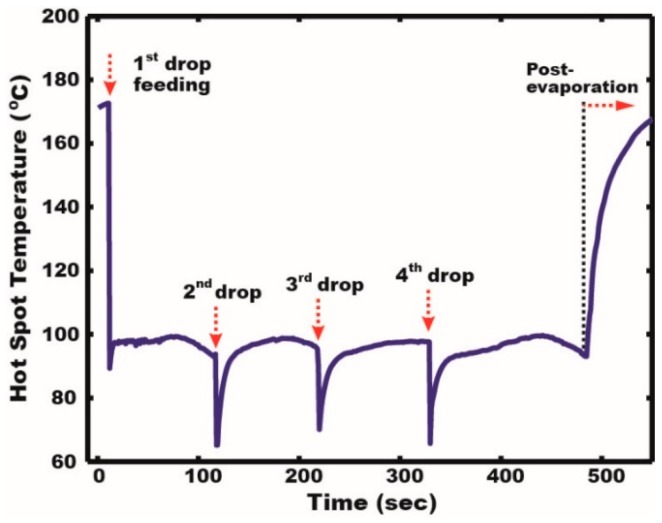
Continuous thermal mitigation by delivering multiple coolant droplets on SDMF. Droplets were successively transported to the hydrophilic hot spot and mitigated the temperature below 100 °C.

Continuous thermal mitigation of the target heating source was demonstrated on a SDMF platform (Figure 6). The hot spot was initially heated up to 172 °C at the heat flux of 6.9 W/cm^2^. SDMF allowed the transportation of a 30 µL water droplet to the target hot spot and the cooling droplet was firmly immobilized on the hydrophilic surface of the hot spot, as presented in Figure 4. Upon the arrival, the temperature quickly dropped down to 89.5 °C (i.e., an instant temperature drop is about 81.5 °C) within a second. While the droplet experienced the two-phase heat transfer (i.e., evaporative cooling), the temperature was maintained below 100 °C. Before the first droplet was fully vaporized, the second droplet was quickly delivered and combined with the liquid remaining on the hot spot. An additional temperature drop of around 29 °C occurred. These processes were repeated with successive delivery of four water droplets and the hot spot temperature was able to be mitigated below 100 °C for 475 s. To mitigate the hot spot temperature even lower, it is suggested to use a cooling liquid that has a boiling temperature lower than that of water. We interestingly observed the instant temperature drop and recovery, when a new droplet was delivered and combined with the liquid puddle on the hot spot. This is because the droplet induces an internal circulation when it is merged with the remaining liquid. This convective flow inside the cooling liquid further enhances the thermal rejection capability of SDMF. Another interesting observation was the temperature decrease of the hot spot before the new droplet arrives. During the evaporation process, coolant liquid keeps losing its mass and thus the liquid layer becomes thin on the hydrophilic hot spot. Such a thin layer of liquid further lowers thermal resistance to improve heat transfer performance, as discussed in the previous section. SDMF would be an effective cooling platform capable of stably supplying the tiny cooling droplet in the range of ~nL to ~µL without additional mechanical components. Such a small volume of coolant liquid forms a thin layer of the liquid, particularly on a super-hydrophilic hot spot surface and allows thin-film evaporation/boiling heat transfer.

### 4.3. High-Heat-Flux Thermal Management

**Figure 7:**
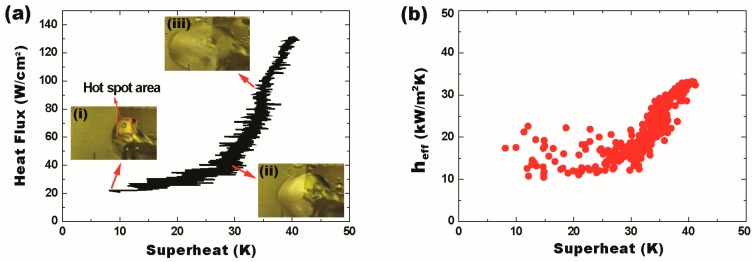
High-heat-flux thermal modulation by the SDMF platform. (**a**) Coolant droplets keep being injected and delivered to cool down the superheat of the target hot spot as low as 40 °C at the heat flux of 131 W/cm^2^. The insets represent the video snapshots of the nucleate boiling process at different heat flux regions of (**i**) 20 W/cm^2^; (**ii**) 45 W/cm^2^; and (**iii**) 90 W/cm^2^; (**b**) The SDMF approach is able to stably manage the effective heat transfer coefficient at a high heat flux regime over 130 W/cm^2^.

Unlike the DMF-based cooling approach, SDMF allows two-phase heat transfer to be used for high-heat-flux thermal management. For the experimental demonstration, a LabVIEW-based voltage control system increased the heater power at the rate of 0.025 V/s to simulate the heat flux increasing on the hot spot. It also allows the continuous injection and delivery of coolant droplets with the volume of 6.7 µL to prevent overheating and flooding. Figure 7 shows the heat flux with the temperature measured at every second. Below the heat flux of 20 W/cm^2^, the superheat varied slightly up and down. As observed in Figure 6, new droplets were combined with the liquid remaining on the hot spot and the circulation flow inside the liquid caused the temperature fluctuation around 2–4 °C. At the heat flux of 20 W/cm^2^, a bubble nucleation was initiated at the surface (an inset image (i) in Figure 7a). Whilst increasing the heat flux, the nucleate boiling was gradually increased and the bubble was released from the surface. To further mitigate the hot spot temperature, the frequency of the droplet supply was increased. Similarly, the delivery of new droplets made the temperature fluctuation around 4–8 °C. Around the heat flux of 45 W/cm^2^, the nucleate boiling became serious and multiple bubbles were generated and released (an inset image (ii) in Figure 7a). Such active bubble motions greatly enhanced the advection flow inside the combined liquid on the hot spot. Sometimes, explosive evacuation of the bubbles popped up a tiny part of the liquid and drove it away from the hot spot location. Above the heat flux of 50 W/cm^2^, the nucleate boiling occurred very intensely while coolant droplets were supplied even faster. Much more bubbles became active and the advection flow was mainly associated with the boiling. An inset image (iii) in Figure 7a shows very intense nucleation boiling around the heat flux of 90 W/cm^2^. Supplementary movie 3 shows evaporation/boiling processes at different heat fluxes. Using the SDMF technology, the superheat was maintained below 40 °C even as the heat flux increased up to 131 W/cm^2^. Due to the power limitation of the thin-film heater, the experiment was stopped to avoid the device burnout.

The effective heat transfer coefficient was estimated as high as 35 kW/m^2^K, even at a high heat flux regime over 130 W/cm^2^ (Figure 7b). Although the heat transfer coefficient does not surpass the values obtained with well-designed recent capillary wicks [31], the SDMF device is able to provide more stable thermal performance especially at a high heat flux regime (>100 W/cm^2^). This indicates that the EWOD-based liquid supply has the potential to outperform the capillary pressure driven supply in terms of the maximum operating heat flux. It is worthwhile noting that both heat transfer coefficient and the maximum heat flux achieved by the suggested SDMF technology can be further improved by reducing the volume of individual droplets, which maximizes the evaporation rate of the droplet in a thin-film form on the super-hydrophobic or hydrophobic hot spot surface. 

## 5. Conclusions

Digital microfluidic (DMF) devices have been recently proposed as an effective on-chip cooling approach. DMF enables rapid transportation of coolant liquid to the target heating sources without any mechanical components such as pumps, microchannels, and capillary wick structures. However, its typical sandwiched configuration with a few hundreds of μm gap only allows sensible heat transfer on a hydrophobic hot spot surface, which significantly limits the thermal rejection capability of the DMF devices. 

To enhance thermal rejection performance, we propose a single-sided digital microfluidic (SDMF) device enabling two-phase evaporative and boiling heat transfer, which is potentially used for high-heat-flux cooling applications. On a single-sided plate, key droplet-based functions necessary for two-phase cooling have been achieved, including continuous droplet injection, rapid transportation as fast as 7.5 cm/s, and immobilization on the target hot spots. While the DMF-based cooling approach mainly uses sensible heat transfer for thermal rejection from a hydrophobic-coated hot spot, our SDMF technology mainly allows two-phase heat transfer (e.g., evaporation and boiling) to reject the heat from a hydrophilic surface of the hot spot. The hot spot surface effect was investigated to quantitatively validate the SDMF’s capability for two-phase cooling. For the hydrophilic hot spot, both the maximum temperature drop and the effective heat transfer coefficient were significantly enhanced and measured to be as high as ~62 K and 33 kW/m^2^K (almost 20 times larger than that of the hydrophobic hot spot), respectively. Continuous thermal modulation based on two-phase heat transfer was experimentally demonstrated with successive droplet delivery. It was also observed that the delivery of new coolant droplets induces an internal circulation inside the liquid remaining on the hot spot, which is able to further improve thermal performance.

High-heat-flux experiments showed that SDMF is able to stably achieve the effective heat transfer coefficient as high as ~35 kW/m^2^K, even at a high heat flux regime over 130 W/cm^2^. Thermal performance on SDMF can be further improved by using different types of coolant liquid that has a lower boiling point. Our SDMF-based cooling approach is potentially applicable for high-heat-flux thermal management based on two-phase heat transfer with the capability of effective coolant delivery on a single-sided plate.

## Supplementary Materials

The following are available online at www.mdpi.com/2072-666X/8/1/3/s1, Video S1: Droplet transportation on SDMF, Video S2: A full set of droplet manipulations for two-phase cooling, and Video S3: High-heat-flux thermal experiment.
